# Lipid Nanovectors to Deliver RNA Oligonucleotides in Cancer

**DOI:** 10.3390/nano6070131

**Published:** 2016-07-09

**Authors:** Virginia Campani, Giuseppina Salzano, Sara Lusa, Giuseppe De Rosa

**Affiliations:** 1 Department of Pharmacy, University Federico II of Naples, Via Domenico Montesano 49, 80131 Naples, Italy; virginia.campani@unina.it; 2 Institute of Molecular Sciences, CNRS, Université Paris-Sud, Université Paris Saclay, 91400 Orsay, France; giuseppina.salzano@unina.it; 3 Department of Biochemistry, Biophysics and General Pathology, Second University of Naples, 80138 Naples, Italy; saralusa@libero.it

**Keywords:** nucleic acid, cationic liposomes, stable nucleic acid lipid particles (SNALPs), lipid-nanoparticles, micelles

## Abstract

The growing knowledge on the mechanisms of gene silencing and gene regulation by non-coding RNAs (ncRNA), mainly small interfering RNA (siRNA) and microRNA (miRNA), is providing a significant boost to the development of new therapeutic strategies for the treatment of cancer. However, the design of RNA-based therapeutics is hampered by biopharmaceutical issues, thus requiring the use of suitable delivery strategies. In this regards, lipid nanovectors have been successfully investigated to deliver RNA in different forms of cancer. Compared to other biomaterials, lipids offer advantages such as biocompatibility, biodegradability, easy production, low cost, limited toxicity and immunogenicity. The possibility to formulate these materials in the form of nanovectors allows overcoming biopharmaceutical issues associated to the therapeutic use of RNA, with the possibility to target tumors. This review takes stock of the main lipid nanovectors proposed to deliver ncRNA. For each considered delivery strategy, the rational design and the most meaningful in vitro and in vivo results are reported and discussed.

## 1. Introduction

### 1.1. RNA Interference and Cancer

RNA interference (RNAi) is a widespread natural phenomenon conserved across different species (fungi, plants, and animals) induced by exogenous RNA oligonucleotides (e.g., small interfering RNA or siRNA) and endogenous small RNA species, such as micro RNA (miRNA) and piwi-interacting RNAs [1]. From the discovery of RNAi by Fire and Mello [2], new mechanisms of gene silencing and gene regulation have been elucidated, providing new tools for biological research and development of new pharmacological strategies. At present, the majority of studies on RNAi are focused on small interfering RNA (siRNA) and microRNA (miRNA). Small interfering RNA are double-stranded 21–25 nucleotide fragments of RNA able to inhibit the expression of specific proteins by inducing the enzymatic cleavage of perfectly complementary target messenger RNAs (mRNAs). Micro RNAs (miRNAs) are endogenous or synthetic fragments of RNA able to bind target mRNAs, modulating gene expression mainly by translation control [1].

Cancer is certainly one of the main targets for RNAi-based therapies. Indeed, oncogenes, mutated tumor suppressor genes, and several other genes involved in tumor progression, are potential targets for gene silencing [3]. Compared to chemotherapy, RNAi-based therapy offers advantages such as a precise functional mechanism, high potential, and high specificity of the gene silencing mechanism. In addition, the use of RNAi in cancer therapy can allow targeting of multiple genes of various cellular pathways involved in tumor progression. Moreover, the RNAi-based therapy can be used to develop personalized drugs for a specific patient [4].

### 1.2. RNA Biopharmaceutical Issues and Therapies

The rapid progresses in the field of RNAi mechanisms and the comprehension of the pivotal role of non-coding RNA (ncRNA) in cancer development and progression has giving rise to positive expectations concerning the discovery of new therapies based on ncRNA molecules, for different forms of cancer. However, the development of RNA-based therapies is hampered by serious drawbacks [5]. Nucleic acids are rapidly degraded by nucleases in biological fluids with very short half-lives. Once incubated in serum or human plasma, only the 25% of the initially-added siRNA was found, while a negligible amount was found detected after 1 h [6,7]. In in vivo experiments, five minutes after intravenous administration of radiolabeled RNA oligonucleotide, no parent compound, but only radiolabeled nucleosides resulting from nuclease hydrolysis, was detected in plasma [8].

Chemical modifications of the RNA backbone can increase the RNA stability in the presence of nucleases, although different variables, such as the type of chemical modification (phosphorotioate, 2′-floride, 2′-*O*-methyl, etc.), the number of modified nucleotides, and the position of modified nucleotides on the strand (5′-modified, 3′-modified, or both), have to be taken into account to optimize the interaction with the intracellular target and the consequent knockdown efficiency [7]. However, an altered interaction with the target, and consequent side effects, should be carefully evaluated when introducing chemical modifications of the RNA chemical backbone. Different biodistribution profiles have been reported for ncRNA following systemic administration, depending on the labeling and on the detection methods. SiRNA conjugation with a chelating agent, diethylenetriaminepentaacetic acid (DTPA), and subsequent labeling with ^111^In, showed the the highest siRNA accumulation in kidneys and highest elimination by renal route. In particular, an average siRNA concentration 40 times higher in the kidneys than in other tissues (brain, muscle, liver, intestine, lung, spleen, and blood) was found [9]. However, the conjugation of the ncRNA with dye, fluorescent dye, or a chelating agent such as the DTPA, change the chemical characteristics of the nucleic acid, with possible influence on the biodistribution profile. Indeed, a different biodistribution was observed by other authors using siRNA radiolabeled with ^3^H [8]. In particular, following 10 min from the i.v. administration, a high concentration in the kidney (7–8 time higher than in the blood) was also found, although similar to that found in other organs (e.g., salivary glands, liver, and spleen). These data suggest that the inclusion of radiolabeled nucleotides remains the preferable approach compared to other labeling methods. It is worthy of note that, when using ^3^H-labeled oligonucleotides, only radiolabeled nucleosides were found in the various tissues suggesting that a rapid siRNAs metabolization and a distribution profile that could be ascribed to small molecular weight metabolites [8].

Another biopharmaceutical issue of ncRNA is their low uptake into the cells, due to the high molecular weight and the polyanionic nature of nucleic acids. Thus, siRNA and miRNA cannot cross the cell membrane, but require entry by endocytosis [5], being that their target is located in the cytoplasm. Viral vectors, such as Lentivirus, Adenovirus, and Herpes Simplex virus, have been proposed for efficient delivery of ncRNA, although the toxicity and the possible immune response, as well as the high cost of the therapies, make difficult the large use of this strategy [10,11]. Non-viral vectors represent a safer and cheaper alternative to the viral vectors. In particular, nanotechnology-based non-viral vectors have been designed to overcome biopharmaceutical issues associated to the therapeutic use of nucleic acids. The use of nanotechnology can allow increasing biological stability of ncRNAs, thus prolonging their half-life. Moreover, nanovectors can be engineered to obtain accumulation in tissues characterized by sinusoidal capillaries (i.e., in the liver, spleen, etc.) or in tumors characterized by altered permeability of the capillaries and lack of lymphatic drainage (the enhanced permeability and retention or EPR effect) [12]. Indeed, these characteristics of the tumors favor the extravasation and the accumulation of nanocarriers in the neoplastic tissues [12]. Plasma components, also called opsonins, can interact with the carrier surface (opsonization) to promote the clearance of the nanocarriers by the cells of the reticuloendothelial system (RES), with a consequently short half-life. The use of a hydrophilic shell, e.g., polyethylene glycol (PEG), on the nanocarrier surface prevents the opsonization, prolonging the circulation time into the blood [13]. The surface of nanovectors can be modified with specific ligands to target receptors over-expressed on the tumor [14]. Among the biomaterials proposed to develop non-viral vectors for the delivery of ncRNA, lipids are certainly the most investigated. Lipid components used to prepare nanocarriers can be biodegradable and biocompatible. Formulations based on lipid nanovectors are relatively easy to develop, also for large-scale production, as testified by different products already in clinical practice. Moreover, varying lipid composition can allow obtaining the required nanovector size, membrane stability, in vivo interactions, and drug release properties [15]. In this review, we provide an overview on the milestones, as well as on the most recent advancements, on lipid-based nanovectors for the delivery siRNA and miRNA for cancer treatment.

## 2. Lipid Vesicles

### 2.1. Cationic Lipids

**Figure 1:**
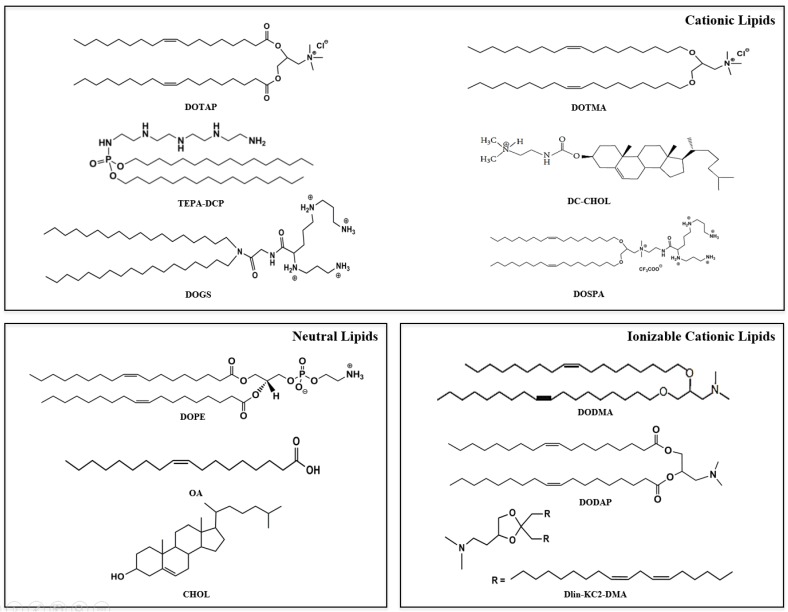
Chemical structure of some lipids commonly used to prepare lipid nanovectors used to deliver ncRNA.

Among the non-viral vectors for drug delivery, lipid vesicles, especially liposomes, are certainly the most investigated. These carriers are based on lipids, mainly—but not exclusively—phospholipids, organized in bilayers that enclose an aqueous phase. Although many types of lipids can be used to prepare liposomes, vesicles based on cationic lipids, namely cationic liposomes (CLs) are certainly the most used to deliver nucleic acid [16]. In fact, the presence of a net positive charge on the carrier surface allows a spontaneous interaction with the negatively-charged nucleic acid, with consequent formation of complexes, also termed lipoplexes [17,18]. The success of this approach can certainly be attributed to the low encapsulation efficiency generally obtained by encapsulating hydrophilic molecules into lipid vesicles. On the other hand, when using cationic lipids, very high complexation efficiencies can be easily achieved. Among the cationic lipids, *N*-[1-(2,3-dioleyloxy)propyl]-*N*,*N*,*N*-trimethylammonium chloride (DOTMA) and 1,2-dioleoyl-3-trimethylammonium-propane (chloride salt) DOTAP are largely the most used to prepare CLs (Figure 1). The presence of a biodegradable chemical ester bond assures DOTAP biodegradation and, consequently, more transient toxic effects related to CLs [19]. Other cationic lipids largely used to prepare CLs and used to deliver ncRNA are dioctadecylamidoglycylspermine (DOGS), 3β[*N*-(*N_*,*N*_-dimethylaminoethane)-carbamoyl] cholesterol (DC-chol) and 2,3-dioleyloxy-*N*-[2(sperminecarboxamido)ethyl]-*N*,*N*-dimethyl-l-propanaminium trifluoroacetate, (DOSPA). Among the above-mentioned cationic lipids, DOSPA and DOGS are multivalent cationic lipids characterized by the presence of different ammonium groups, which contribute to a more efficient packing and binding of nucleic acids, compared to monovalent cationic lipids [20]. The complexation with CLs protects the nucleic acid toward the enzymatic degradation, thus improving their stability in biological fluids [18,21,22].

### 2.2. Intracellular Delivery of ncRNAs

The ability of lipoplexes to promote the delivery RNA oligonucleotides into the cells can be considered a well-consolidated concept [23,24,25]. First evidence of in vitro “lipofection” with RNA was provided by Malone et al. who used DOTMA/DOPE-CLs (Lipofectin**^®^**) to improve RNA transfection into cells. In this study, the Lipofectin/RNA weight ratio of 2.5 was found to provide the highest transfection efficiency, corresponding to 70% of the transfected cells. In the same study, Lipofectin was used to successfully transfect a wide variety of cells, namely human, mouse, rat, *Xenopus*, and *Drosophila* cells, although with a high variability of the transfection efficiency [24]. When using lipoplexes, the number of experimental variables (+/− charge ratio, lipoplex concentration, maturation time of the lipoplexes before use, presence and concentration of serum, cell type, etc.) leads to high variability of the transfection efficiency. The influence of some experimental parameters when preparing DC-chol lipoplexes to achieve the highest siRNA delivery efficiency has been addressed by Zhang et al. [25]. In this study, the highest siRNA transfection efficiency was found at high DC-chol/siRNA weight ratios, up to about five or 10, with a plateau for a further increase of the weight ratio.

The entrance of lipoplexes into the cells is mediated by the “clathrin-mediated endocytosis” [17,26]. The interaction of lipoplexes with cells is regulated by the electrostatic interactions between the positive charge of the lipoplexes and the negative charges of the cells membrane. The escape of nucleic acids from endosomes occurs through the destabilization of the endosomal membrane due to “flip flop” movements of the lipids. In particular, the anionic lipids, mainly located on the cytoplasmic face of the endosomes, diffuse into the complex neutralizing it; the oligonucleotide is consequently displaced from the cationic lipid and is free to diffuse into the cytoplasm [27]. Chemical modifications on the cationic lipids, namely the use of lipids with different hydrophobic chain lengths and saturation degrees, or the chemical modification of the head group structure, have been proposed to improve the transfection efficiency of CLs. Thus, starting from the most commonly used cationic lipids, e.g., DOTMA or DOTAP, novel cationic lipids have been developed to increase the transfection efficiency of nucleic acids. Indeed, cationic lipids with shorter hydrophobic or longer unsaturated tails, as well as with modified charged head group, have been proposed. In the multivalent cationic lipids DOSPA and DOGS (Figure 1), the introduction of the spermine group allowed persistence of the positive charge improving lipoplex endocytosis and, consequently, the transfection efficiency [16,27]. As mentioned above, CL formulations generally also include neutral lipids in the bilayer, also termed as “helper lipids”. Among them, cholesterol (chol) can improve CL complexation with nucleic acids [28], although an excess of this neutral lipid was found to be detrimental for the liposome formation. The influence of chol [29] when associated to the cationic lipids, has been largely reported. Chol has been used to improve the transfection efficiency of nucleic acids with CL, although this effect has been attributed to the enhanced physical stability of the colloidal dispersion [30,31,32,33]. Dioleoylphosphatidyl ethanolamine (DOPE) (Figure 1), due to its fusogenic properties, is certainly the helper lipid most frequently used in the preparation of CLs. Once at the acidic pH of the endosomes, DOPE present in the CL bilayer segregates from the lipid mix, shifting from a lamellar structure to an inverted hexagonal phase on which nucleic acid condenses with cationic lipids to form a honeycomb tubular shape; in these conditions, lipoplexes fuse with the endosomal membrane, with consequent release of the nucleic acid into the cytoplasm [34,35]. However, the optimal cationic lipid/DOPE ratio should be investigated for each cell type, cationic lipid, and when changing the experimental conditions. Zhang et al. found the highest siRNA transfection efficiency with DC-chol/DOPE used at the molar ratio of 1 [25]. Additionally, the use of hydrogenated soya phosphocholine (HSPC) was found to increase the complexation strength between DOTAP liposomes and siRNA [28]. This has been attributed to the separation of cationic charges on the liposome surface, in the presence of HSPC in between the DOTAP molecules [20,36]. Oleic acid (OA) has also been proposed as helper lipid in RNA-containing lipoplexes, to confer pH-sensitivity, smaller particle size, and surface charge close to neutrality [37]. The polycation dicetyl phosphate-tetraethylenepentamine (TEPA-PCL) has also been proposed to replace cationic lipids in the liposomes for the delivery of RNA oligonucleotide In particular; in vitro experiments carried out on human umbilical vein endothelial cells (HUVECs) demonstrated that complexation of miR-92a with TEPA-PCL liposomes improved the delivery of miRNAs with an enhancement of the cellular-uptake and endosomal escape of miR-92a, if compared to CLs liposomes. These findings were attributed to the proton sponge effect of TEPA-PCL [38]. Liposomes containing the polycation dicetyl phosphate-diethylenetriamine (DCP-DETA) have also been proposed by Koide et al. to deliver siRNA [39]. They reported that the DOPE/chol/DCP-DETA CLs were firstly compexed with siRNA and then freeze-thawed. Following freeze-thawing of a single-layer CLs/siRNA complex, a “packed multi-layer structure” is formed, suggesting that siRNA was effectively encapsulated between the lipid layers. Interestingly, no degradation of encapsulated siRNA in freeze-thawed lipoplex was found in bovine serum for 72 h, while, in the same experimental conditions, 90% of siRNA in the conventional lipoplex was degraded [39].

The authors have tried to show how the variation of the vesicle structure, and in particular the lipid composition, can strongly influence and lead to improved RNA delivery into the cells. Moreover, the obtaining of a successful RNA delivery system shall take into account multifactorial aspects. Different experimental parameters have been identified to affect lipoplex characteristics as well as the transfection efficiency. The balance of the +/− charge, as well as the ratio between the lipids, can influence the entity of transfection [16,17]. A high CL/siRNA weight ratio, e.g., of 7.5, was needed to completely retard siRNA in a gel retardation assay. However, with the same technique, the introduction of a PEGylated lipid into the DC-chol/DOPE liposomes showed a significant reduction of siRNA complexation efficiency. Finally, the same authors hypothesized that DOPE also has binding affinity to nucleic acids, although weak, due to the highest siRNA binding affinity found in complexes based on DC-chol/DOPE CLs, compared to DOPE-free [25] complexes. Additionally, the size of CLs, as well as the incubation medium in which lipoplex are prepared, can significantly influence the transfection efficacy [17,40]. In vitro experiments on human osteosarcoma cell line (HOS) evidenced that the preincubation of DOTMA/DOPE CLs with phosphate buffer strongly reduced the time for cellular uptake of RNA, as well as the time to transfection. The authors of the study hypothesized that divalent phosphate anions could favor fusion of cationic lipids and consequently promote fusion with the cell membrane [40]. Moreover, it has been demonstrated that the agitation during siRNA/CL complex formation results in a superior gene knockdown efficacy. It has been hypothesized that a constant and intense agitation limits the interactions between the cationic lipid and DOPE, while promoting the interactions between cationic lipid and siRNA, with a consequent higher complexation efficiency and an increased gene knockdown effect [41]. The composition of CLs and the structure of a cationic lipid can influence not only the transfection efficiency of the resulting lipoplexes, but also their cytotoxicity [42]. CL cytotoxicity has been mainly associated to the net positive charge of the polar head group, to the linker, and to the hydrophobic domains of cationic lipids [43,44]. It has been reported that, at low concentrations, cationic lipids can affect the exposed cell and cause cytoplasm vacuolization, reduce the number of mitoses, and induce cell constriction. On the other hand, at high concentrations, the surfactant activity of cationic lipids can alter the integrity of the membrane structure, thus causing cell lysis and necrosis [19,42,45]. Furthermore, the interaction of the cationic groups with cellular enzymes such as protein kinase C (PKC) can contribute to the cell toxicity [43]. In line with this, cationic lipids with a quaternary ammonium amphiphilic head groups showed a higher toxicity compared to cationic lipids containing tertiary amines [46]. Moreover, the delocalization of net positive charge by introduction of a heterocyclic ring to replace the linear amine head group, can decrease the cytotoxicity of the cationic lipid [47]. On the other hand, it has been demonstrated that cationic lipid with a hydrophobic double-tail chain are less toxic than their counterpart with a single-tail aliphatic chain (e.g., DOTMA was found to be less cytotoxic than the cationic lipid cetyl trimethylammonium bromide) [48]. Finally, the cationic lipid containing steroid backbones (cholesterol derivatives) were more potent inhibitors of PKC, thus resultingly more toxic than their straight-chain analogues [46]. The nature of the linker group between the cationic head group and the hydrophobic tail is also held responsible for the in vitro toxicity of CLs. In fact, as mentioned above, cationic lipids such as DOTMA with a stable ether linker group are more toxic compared to cationic lipids with biodegradable and more feeble ester groups, such as DOTAP [42]. Another approach to reduce the cytotoxicity associated to the CLs is to introduce negatively-charged lipids in the formulation to reduce the net positive charge of the final lipoplexes. In accordance with this, dimyristoyl-*sn*-glycero-3-phosphoglycerol (DMPG) was used to neutralize the net surface charge of DOTAP-based CLs [27], although with a lower RNA complexation efficiency. CLs have also shown hemolytic properties, attributed to the cationic charges present on the vesicle surface that lead to the formation of pores on the cell membrane [44]. CL complexation with siRNA reduced the hemolytic properties, probably for the reduction of the free positive charge. Moreover, the use of PEGylated lipids, lead to a further reduction of the lipoplex hemolytic activity, probably due to the further shielding of the positive charges [36]. 

Thus, taking into account all of the previously-described findings, the formulation of CL for ncRNA should be a compromise between an efficient RNA complexation, limited cell tocixity and hemolytic properties. Helper lipids can be considered to promote the RNA escape from the endosome, but also to modulate the RNA complexation efficiency. Finally, the use of PEGylated lipids reduces the complexation efficiency, but it can be mandatory to make these formulations less toxic, and more stable in serum, thus allowing the in vivo use of these carriers (see below).

### 2.3. Local and Systemic Delivery of ncRNA

The preparation procedures of lipoplexes, obtained by RNA complexation before use, encouraged the in vivo use of these formulations. Once that the preparation conditions have been fixed, lipoplex preparation can be relatively easy. This certainly contributed to the large use of lipoplexes in vivo. In vivo lipid-vesicles for RNA delivery can be administrated locally or systemically. The intratumoral injection of lipid nanovectors assures a high bioavailability with a reduced toxicity, compared with a systemic administration. This approach has been proposed for the treatment of more accessible tumors, such as melanoma [49]. Garbuzenko et al. compared the intratracheal and the intraveneous administration of DOTAP-CLs encapsulating siRNA in an orthotopic mouse model of human lung cancer. The local administration allowed achieving siRNA-containing lipoplexes into the tumor at higher concentration and for longer timeframes (up to three days). The intratracheal administration also resulted in higher inhibition of the tumor growth when compared with systemically-administered lipoplexes [50]. CLs were also successfully used for topical delivery of BRAF-targeted siRNA to melanoma cells located into the basal epidermis [51]. In this study, the authors included in the formulations a penetration enhancer, i.e., sodium cholate (NaChol), already proposed by other authors [52] to open pores in the stratum corneum, thus accelerating permeation of nanoparticles through the skin. As already discussed in the previous paragraph, an efficient delivery system for ncRNA can be obtained by an optimized formulation. Thus, different formulations containing DOTAP-CLs, at different NaChol/DOTAP and CL/siRNA ratios, were prepared. The authors demonstrated that a correct balance of lipoplex size, charge, and content of the edge activator is required for an efficient siRNA delivery/deposition through/into the skin. In particular, DOTAP/NaChol CLs, weight ratio 8:1, complexed with siRNA at a RNA/CL weight ratio of 16:1, allowed the highest accumulation of siRNA in the deeper skin layers, i.e., dermis, compared to other CL formulations. Moreover, all lipoplexes could effectively internalize into melanoma UACC-903 cells, although the highest internalization and localization in the cytoplasm, associated to a significant reduction of BRAF protein expression, was achieved with the optimized formulation [51].

An important issue to consider, especially when using lipoplexes by intravenous administration, is their instability in the presence of serum components [17,42]. When systemically administrated, lipoplexes interact with the negatively-charged serum proteins, e.g., albumin [53]. This led to lipoplex aggregation, reduction of their half-life, and consequent accumulation in some filtration organs, such as the spleen [42,54,55]. It has been reported that the presence of serum can lead to a significant reduction of transfection efficiency compared to in vitro experiments on cells treated with serum-free media (e.g., NaCl solution). Indeed, serum protein could interfere with the interaction of the lipoplexes with the cells, also causing decomplexation, release, and degradation of nucleic acids [17]. Thus, different strategies have been tested to reduce serum susceptibility of lipoplexes. It has been demonstrated that the introduction of helper lipids such as DOPE or chol can stabilize the carrier membrane, reducing vesicle aggregation in the presence of serum proteins [17]. It is worthy of note that in vivo CLs are relatively toxic carriers and can trigger an immunological reaction with the production of antibodies; they can also induce systemic inflammation as consequence of the incorporation of CLs by the Kupffer cells of liver [19,56,57]. Thus, PEG moieties have been introduced on the membrane of CLs (generally by using PEGylated phospholipids) to increase the stability of lipoplexes in biological fluids and to reduce toxicity and immunological reaction following systemic administration. PEG moieties on the surface of the liposomes lead to the formation of a steric barrier around the lipoplexes, preventing the aggregation of the carriers. Thus, PEGylation of CLs reduces the macrophage uptake, increases the bioavailability, and prolongs the circulation time in the blood. Consequently, PEGylation of RNA-containing lipoplexes favors the transfection efficiencies due to the improved tissue distribution and to the larger available concentrations [19,20]. It is also noteworthy that PEGylation has drawbacks, such as the production of anti-PEG-IgM, which is responsible of the accelerated blood clearance (ABC) phenomenon responsible for the more rapid clearance of the nanovectors following the first administration [58,59]. Lung is certainly a preferred accumulation site for lipoplexes. Thus, different studies have proposed the use of CL to deliver siRNA or miRNA to the lung to treat lung cancer. PEGylated CLs, composed of DOTMA, chol, and D-Alpha-tocopheryl polyethylene glycol 1000 succinate, have been designed to deliver miR-133b in ung cancer [60]. By using this formulation, ~30% of miR-133b accumulated in the lung four hours post intravenous injection [60]. Wu et al. successfully used the same formulation for the delivery of miR-29b into the lung by intravenous injection for the treatment of lung cancer. In this study they also demonstrated that lipoplexes efficiently deliver miR-29b in non-small cell lung cancer (NSCLC) tissues, with significant inhibition of tumor growth [61]. It has been hypothesized that the preferential lipoplex accumulation in the lung should be due to the highly positively-charged DOTMA lipoplexes, which prevent accumulation in the liver [60]. The inclusion of helper lipids, able to shield the positive charge of lipoplexes, could be used to reduce accumulation in the lung, shifting the delivery in other organs, among them, the liver. The inclusion of OA in PEGylated cholesterol/DOTMA-based CLs was investigated for the delivery of miR-122 in the liver, for replacement therapy in the treatment of liver cancer. Thus, in an experimental model of liver cancer, intravenously-injected OA-containing lipoplexes accumulated in the liver, where levels of miR-122 were found significantly higher compared to that found in other organs, such as the spleen and kidney. Moreover, no accumulation of miR-122 was found in the lung and heart. Moreover, no toxicity was reported following the administration of the lipoplexes. Interestingly, in the same study, DOTMA-CLs (without OA) accumulate in the lungs, suggesting that OA, used as helper lipid in the CLs bilayer, can also be useful to change lipoplex biodistribution and to target the liver [61].

An evolution of nanomedicine is the development of a carrier able to target specific cells. In line with this, the addition of specific ligands, i.e., monoclonal antibodies peptides, aptamers, and oligosaccharides has been proposed to increase the affinity of lipoplexes towards specific cells, as well as to improve the RNA transfection efficiency. Lee et al. developed targeted cationic liposomes based on DOTAP and cholesterol for the delivery of let-7a-miR in lung cancer. The surface of the CLs was decorated with the ligand Eph-A1 through the cyanur groups linked to the 1,2-distearoyl-sn-glycero-3-phosphoethanolamine-*N*-[amino(polyethylene glycol)-2000] (DSPE-PEG) lipid. The transfection of let-7a-miR in lung cancers cells was higher compared to untargeted lipoplexes [62]. Additionally, albumin has been used as a ligand for targeting. Indeed, Passadouro et al. proposed CLs composed of 1-palmitoyl-2-oleoyl-sn-glycero-3-ethylphosphocholine (EPOPC) and cholesterol for the complexation of anti-microRNA (against miR-21, miR-221, miR-222, and miR-10 overexpressed in pancreatic ductal adenocarcinoma cells, PDAC) associated to human albumin to target PDAC. Both in vitro and in vivo, the use of albumin led to a higher transfection efficiency of lipoplexes on PDAC, with consequent inhibition of miRNAs expression. In addition, the association of albumin-CLs-anti-microRNA with the chemotherapeutic agent sunitinib resulted in a synergism between anti-miRNAs and sunitinib, consequently reducing the required active dose of the chemotherapeutic agent [63]. Moreover, Meissner et al. designed lipid-nanocarriers for targeted delivery of anti-BCL2 miRNA. In fact, the overexpression of the antiapoptotic gene BCL2 in leukemia cells overexpressing the protein CD20 has been evidenced in patients affected by leukemia. In order to regulate the expression of the BCL2, DOTAP/anti-BCL2 miRNA lipoplexes, or polyethylenimine (PEI)/anti-BCL2 miRNA complexes were encapsulated in liposomes with anti-CD20 antibodies (Rituximab), linked to the surface of the vesicles through the maleimide groups present on a PEG-lipids. The formulations efficiently delivered the anti-BCL2 miRNA in the cells overexpressing CD20, leading to a significant reduction of BCL2 protein expression. In vivo studies on NOD/SCID mice inoculated with the Daudi human Burkitt’s lymphoma cell evidenced that both the formulations strongly inhibited the tumor growth in mice, leading to a completed remission of the tumor mass [64].

These studies demonstrated that the possibility to use lipoplexes in vivo to target tumors. The use of ligands for a more selective delivery to target cells can increase the efficacy of the therapy and reducing the possible side effect due to transfection of healthy cells.

### 2.4. Stable Nucleic acid Lipid Particles

Starting from CLs, more advanced lipid vesicles, namely stable nucleic acid lipid particles, were designed (Table 1). Stable nucleic acid lipid particles (SNALPs), introduced by Semple and colleagues about 15 years ago [65], can be considered an evolution of CLs for the delivery of nucleic acids. A standard SNALP formulation is characterized by a high transition temperature phospholipid, e.g., 1,2-distearoyl-sn-glycero-3-phosphocholine (DSPC), a PEGylated lipid and an ionizable cationic lipid, e.g., 1,2-dioleyloxy-*N*,*N*-dimethyl-3-aminopropane (DODMA) or 1,2-dioleyl-3-dimethylammonium propane (DODAP) (Figure 1) [66]. The positive charge of DODAP allows achieving high RNA encapsulation efficiency; however, the net charge exposed on the surface of the vesicles can be neutralized after RNA encapsulation, making these vesicles more stable in biological fluids [65], compared also to CLs. In the last ten years, many ionizable cationic lipids have been synthetized [66]. For example, starting from DODMA, different cationic lipids with different degrees of saturations, i.e., 1,2-distearyloxy-*N*,*N*-dimethyl-3-aminopropane (DSDMA), 1,2-dilinoleyloxy-*N*,*N*-dimethyl-3-aminopropane (DLinDMA), or 1,2-dilinolenyloxy-*N*,*N-*dimethyl-3-aminopropane (DLenDMA), were synthetized to improve their fusogenic properties [67]. Thus, it has been evidenced that, with the increase of the number of double bonds, there is a decrease of the phase transition temperature from the lamellar to the hexagonal structure, with a significant improvement of the transfection efficiency [67]. Among different ionizable cationic lipids, DLinDMA (Figure 1) showed the highest transfection efficiency when used in SNALPs, which were also tested on human primates and in clinical studies [66]. Thus, starting from DLinDMA, a rational design was carried out by changing the headgroup and the linker to synthesize a lipid derivative with the best-performing lipid to prepare SNALPs. Thus, the use of nanovectors based on the newly synthesized ionizable lipid, namely DLin-KC2-DMA, improved the in vivo activity of siRNA at doses of 0.01 mg/kg in rodents and 0.1 mg/kg in non-human primates [68,69]. The addition of DSPC in the lipid membrane was needed to confer stability to the lipid bilayer during the vesicle preparation and during the circulation. SNALPs for the delivery of siRNA and miRNAs in different tumors have been proposed [70,71,72] with some SNALP-based formulations currently in clinical trials. Finally, PEG could also be used to decorate the surface with ligands, in order to develop targeted SNALPs. Our research group developed targeted-SNALPs to deliver miR34a in multiple myeloma cells. The conjugation of SNALP encapsulating miR34a with transferrin resulted in a higher inhibition of tumor growth and longer mice survival, compared to untargeted SNALPs. Moreover, a further increase of mice survival was observed when a chemically-modified miRNA, and not a wild-type miRNA, was used [73]. In another study, the conjugation of chlorotoxin on the SNALP surface resulted in an improved anti-miR-21 internalization in GL261 mouse glioma cells; the systemic administration of chlorotoxin-SNALPs-anti-miR-21 induced expression of the anti-miR-21 target RhoB. Finally, in an orthotropic model of glioblastoma, chlorotoxin-SNALPs, in association with the chemotropic agent sunitinib, led to an enhanced reduction of the tumor mass and to an improvement of mice survival [74].

Altogether, these results demonstrate that SNALPs can be considered an evolution of cationic lipids, overcoming some issues associated to the latter. Indeed, differently than lipoplex, physical characteristics of SNALPs are quite independent on the preparation conditions, while maintaining the transfection efficiency of the CLs.

## 3. Self-Assembled Core/Shell Lipid Nanoparticles

Core/shell lipid-based nanovectors have been proposed to deliver siRNA and miRNA. The research group of Huang gave a significant contribution to the development of this strategy by developing nanoparticles obtained by RNA complexation with polycation, then covered by a lipid shell. More in detail, in the first studies, RNA (previously mixed with calf thymus DNA at the 1:1 weight ratio) was condensed into the NP core by mixing with protamine, a highly positively-charged peptide already used as transfection reagent for nucleic acids. The high molecular weight DNA was reported to improve the core compaction, while the use of calf thymus DNA, compared to plasmid DNA, was preferred for the limited amounts of immunostimulating CpG motifs [75]. The resulting RNA-containing complexes were covered with a lipid shell by mixing with cationic liposomes (DOTAP/chol) to provide liposome-polycation-DNA (LPD) nanoparticles. To reduce the particle size and to increase the stability in serum (thus preventing the NP aggregation), LPD NPs were mixed with DSPE-PEG by post-insertion method [76]. It is worthy of note that PEGylation introduced steric hindrance to LPD, thus reducing about 80% of their delivery efficiency; the use of ligands, e.g., anisamide, linked to the DSPE-PEG restored the delivery efficacy of these NPs. Anisamide, a small molecule to target human lung cancer cells also allowed achieving significant improvement of LPD localization in lung cancer [76]. In different animal models of cancer, siRNA, when encapsulated in LPD NPs, showed an increased circulation time compared to free siRNA; no differences were found between targeted and untargeted NPs [75]. Interestingly, NPs were cleared from the blood significantly quicker in tumor-bearing than in healthy mice. Moreover, NPs increased the half-life of the siRNA in the blood, increased the area under the curve and decreasing the clearance [76]. About 80%–70% of the injected siRNA accumulated into the tumor by using LPD NPs, without significant differences between targeted and untargeted NPs [75,76]. Interestingly, in the tumor tissue, the targeted NPs allowed an increased siRNA delivery into the cytoplasm, compared to untargeted NPs. This technology has also been used to successfully deliver a mixture of siRNAs against different targets to obtain simultaneous silencing of three oncogenes, with consequent significant inhibition of tumor growth and prolonged mice survival in a model of lung metastasis [76]. Taking into account the capability of doxorubicin (DOX) to interact with double stranded DNA by non-covalent intercalation, DOX was also co-encapsulated with siRNA for combined therapy [77]. The replacement of calf thymus DNA with hyaluronic acid (HA) into the core of the NPs (LPH NPs), allowed reducing the production of pro-inflammatory cytokines [78]. The pro-apoptotic effect of siRNA-encapsulating NPs can be enhanced by replacing DOTAP with a newly-synthesized, non-glycerol-based trivalent cationic lipid, namely DSGLA [79]. The authors also demonstrated that, when adding the DSPE-PEG to the NPs, a large percentage of lipids was stripped off by the DSPE-PEG and formed smaller particles (mean diameter lower than 100 nm). In particular, about 90% of the siRNA used in the formulation was associated to approximately 37.2% of the total lipid and 20.2% of the input DSPE-PEG. In the same study, the authors demonstrated that the arrangement of PEG in the brush mode on the NP surface prevented opsonization in serum and abolished the non-specific uptake by RES in the isolated liver [80]. LPH NPs, targeted with the single-chain antibody fragment (scFv), have also been used to simultaneously deliver siRNA and miRNA (miR34a) [77]. To increase the siRNA delivery, HA and protamine were replaced by calcium/phosphate (CaP) NPs to complex siRNA in the core of the lipid-coated NPs or LCP NPs [75]. The authors hypothesize that LCPs NPs can enter by endocytosis and disassemble into the endosomes for the lower pH; this should induce an increase of the endosome intracellular pressure and swelling, with consequent release of the entrapped siRNA [75]. This technology has been successfully used to simultaneously deliver siRNA against three different targets in different experimental models of tumor [81,82]. The technology proposed by Huang and colleagues certainly represent a valid alternative to lipid vesicles, although these two delivery systems were never compared in the same experimental conditions. As in the case of SNALPs, the core/shell NPs described above appear as a versatile delivery system, quite independent on the experimental condition of use. A scale-up process could evaluate if core/shell NPs could also be proposed for clinical use. Self-assembled anionic NPs encapsulating RNA have also been proposed. The anionic charge should reduce the toxicity and the rapid clearance from the circulation generally associated to the cationic NPs. Thus, NPs consisting in a mixture of peptide ligands and anionic, PEGylated liposomes were developed [83]. These components, at the optimized molar charge ratios and in the well-established order of mixing, self-assemble into anionic NP with about 80%–91% packaging of siRNA in the absence of serum; in presence of serum, the quenching was only reduced by 1.2 to two-fold [84]. The authors of these studies reported a higher stability of PEGylated anionic nanocomplexes, compared to their cationic counterpart. They also report a more ready decomplexation in presence of heparin, suggesting their potential to dissociate once into the cytoplasm. These NPs were successfully used to deliver the siRNA into the rat brain [84]. The use of cationic formulation has been associate to toxicity at different levels, such as mitochondrial damage, interfering with blood coagulation cascade, induction of interferon response, promotion of cytokine production, and complementary activation [85,86]. Thus, neutral lipid-based nanoparticles (LNPs) have been also proposed to deliver RNA oligonucleotides with a more safe profile. These NPs has also been chemically modified with hyaluronan (Han) to confer stealth properties and to target CD44-overexpressing cancer cells. In detail, these NPs were prepared by the self-assembling of neutral phospholipids and cholesterol. The authors showed a silencing effect only after six days following the transfection, while the same effect was found after two days in the case of lipofectamine used for comparison purpose [84]. The authors attributed this delay to the slow release of siRNA from the endosomes while, in the case of the cationic liposomes (e.g., lipofectamine), the endosomal escape should be enhanced. It is worthy of note that, in this work, in vivo safety data on this formulation has not been provided, especially in comparison with cationic nanovectors. 

## 4. Solid Lipid Nanoparticles

Kim et al. proposed to use solid lipid NPs (SLNs) for the delivery of RNA. SLNs are NPs composed of approximately 0.1–30 (% *w*/*w*) solid fat dispersed in an aqueous phase with the help of surfactants, at a concentration of about 0.5%–5% [85]. The SLN, composed of cholesteryl ester, triglyceride, chol, DOPE, and DC-chol, were prepared by a modified solvent-emulsification method [79]. SiRNA, previously conjugated with PEG, was then mixed with SLN obtaining siRNA-PEG/SLN complexes [86]. The PEG on the surface of siRNA-PEG/SLN complexes prevented nonspecific protein adsorption, resulting in high stability in the serum, and prolonged the NP circulation in the blood stream [86]. At the optimal DC-chol/siRNA-PEG weight ratio, the complexes showed a dose-dependent gene silencing efficiency, associated with a low cytotoxicity [86]. The same system has been proposed to deliver siRNA in an orthotopic model of glioblastoma. Immunohistochemical analysis of the tumor sections after treatment with the formulation containing siRNA, demonstrated the efficacy of the delivered siRNA in downregulating the targeted gene with consequent reduction of the tumor growth; in the same study, no significant change in the body weight in both treated and untreated mice was observed, suggesting the lack of systemic toxicity [87]. The intracranial uptake of siRNA-PEG/SLN complex was also demonstrated by using fluorescently-labelled NPs. An alternative approach to incorporate siRNA in the core of SLN was based on a hydrophobic ion pairing strategy. Briefly, siRNA was complexed with a cationic lipid, DOTAP, and incorporated in SLN based on lecithin and DSPE-PEG. These SLNs showed a prolonged release of siRNA, in vitro and in vivo, over a period of 10–13 days [88]. SLNs have also been proposed for co-delivery of siRNA and chemotherapeutic agents, e.g., paclitaxel (PTX), for combined anticancer therapy. In this study, PTX was associated to the lipid mix, mainly based on DC-chol, retinol, DSPE-PEG, and other phospholipids. An improved anti-cancer efficacy of the SLN encapsulating PTX and complexing siRNA was demonstrated in vitro and in vivo [89]. In the case of the SLNs, the slow release could be an additional value compared to the delivery systems described in the previous paragraphs. Moreover, SLNs do not present scale-up issues and can be sterilized. Finally, the absence of organic solvent in the preparation process make this SLNs especially attractive for future clinical application. 

## 5. Lipid Micelles

Micelles are core-shell supramolecular nanostructures of a diameter in the range of 10–100 nm, formed from the self-assembling of amphiphilic molecules in an aqueous environment. Micelles developed for drug delivery purposes consist of a hydrophobic core, in which poorly water-soluble molecules can be solubilized, and a hydrophilic shell, where polar molecules can get absorbed. Several lipids or di-block and tri-block amphiphilic copolymers can be used to obtain micelles. Lipids, and in particular phospholipids like DSPE, have been used to form a hydrophobic core, [90,91]. PEG is by far the most often used shell-forming polymer, when linked to polymeric or lipid monomers. Micelles exhibit several advantages which favor their wide use for drug delivery in the treatment of various diseases. Among many, their versatility to incorporate both hydrophobic and hydrophilic molecules, the range of size suitable for extended circulation time in the blood, but at the same time avoiding a fast renal clearance and, finally, the easy scale-up of the formulations, make micelles an attractive delivery system.

In the last decade, RNA oligonucleotides, especially siRNAs, have been efficiently incorporated in micelles. To date, three main strategies have been exploited to incorporate RNAs in micelles. The first approach, based on polyelectrolyte complex (PEC), involves as first step the direct conjugation of siRNA to PEG [92] or lipid moieties via degradable or non-degradable linkages followed by their condensation with cationic core-forming agents, including polymers, peptides, or lipids. With this in mind, in a second strategy, an amphiphilic block co-polymer containing low molecular weight PEIs of 1.8 kDa, PEI conjugated to a fusogenic lipid, DOPE, was synthetized to complex siRNAs [93]. Despite the presence of DOPE, a similar complexation efficiency of the siRNA was observed compared to non-modified PEI. The presence of DOPE significantly enhanced the transfection efficiency of PEI, which alone was ineffective. PEG derivative with lipids (PE), PEG-PE, well-known to form stable and biocompatible micelles, were then added to the DOPE-PEI-siRNA complex to confer long circulation proprieties for in vivo administration [94]. The intravenous injection of the PEGylated DOPE-PEI-siRNA resulted in an 8% accumulation of the injected dose in solid tumors. Then, the therapeutic efficacy was assessed in mice bearing multi-drug resistant breast cancer cells, MCF7/MDR. Briefly, mice were intravenously injected once a week with DOPE-PEI/siRNA-mediated P-gp downregulation formulations followed by Dox injection (after 48 h) for five weeks. A significant sensitization of the tumors to otherwise non-effective doses of free Dox was achieved by the combination therapy. Furthermore, when using DOPE-PEI formulations, sequence-specific P-gp downregulation was found by RT-PCR analysis in excised tumors, confirming the efficacy of the developed formulation to safely deliver intact siRNA into the tumor tissues [94]. By the same group, the PEG/DOPE-PEI-siRNA micellar system was upgraded by developing, for the first time, hypoxia-sensitive siRNA micelles [95]. In particular, the conjugate PEI-DOPE was directly linked to a PEG2000 chain via a hypoxia sensitive azobenzene group [95]. The resulting conjugate, namely PEG-azobenzene-PEI-DOPE, in an aqueous environment efficiently complex siRNA directly against a green fluorescent protein (GFP), forming nanosized “core-shell” micelle-like structures. With this strategy, the long PEG chain was used to confer longevity of the whole system when in circulation, once in oxygen-deprived regions the azobenzene group could lead to the detachment of the PEG chain, addressing the well-known “PEG dilemma”. Indeed, it has been widely reported that PEGylation of the nanovector results in a significant suppression of its cellular uptake and the endosomal escape [56]. Interestingly, the developed hypoxia-sensitive siRNA micelles showed a significant cellular penetration of the siRNA with consequent knock down of the levels of GFP in various GFP over-expressing cancer cell lines only under hypoxic condition. In mice bearing A2780/GFP tumor cells, the intravenous administration of the hypoxia-sensitive formulation resulted in a GFP downregulation of about 24% and 32%, found by ex vivo imaging and by flow cytometry, respectively [95].

In a third approach, the direct conjugation of siRNA with lipids followed by its incorporation in PEG-PE micelles was proposed to completely avoid the use of cationic polymers [96]. By a simple reaction, siRNA was directly linked to PE by introducing a stimuli-sensitive linker, a disulfide moiety. The obtained siRNA-S-S-PE conjugate was then efficiently incorporated in PEG-PE through the amphiphilic interaction between the PE moieties of the two conjugates. Stability of the siRNA against enzymatic degradation and an efficient silencing activity in vitro has been achieved [96]. Interestingly, the introduction of the disulfide linkage could release intact siRNA preferentially in cancer cells. It has been reported that, in cancer cells, the concentration of reductase, including glutathione (GSH), is estimated to be up to 1000-fold higher than in the blood; moreover, the extracellular levels of GSH in tumor tissues are 100-fold higher than the ones in healthy tissues [97]. This environmental trigger can be exploited to regulate the release of siRNA in tumor tissues [98]. In fact, the intravenous administration of siRNA-S-S-PE PM containing a siRNA directed against survivin, a protein upregulated in cancer cells and involved in multi-drug resistance, led to a significant downregulation of both protein and mRNA levels of the targeted gene in a mice model of cancer [91]. So far, many interesting micellar-based systems have been designed for siRNAs delivery. “Basic” polycationic micelles have been modified continuously to achieve maximum protection of the siRNA and its release at a desired site by introducing environmentally-sensitive moieties, fusogenic lipids, and specific polymers for endosomal escape. As follows, we discuss some examples of micelles combining multiple functional moieties and containing a combination of conventional drugs and siRNAs, namely multifunctional micelles. 

Engineered micelles able to combine in one delivery system a number of distinct functional components have also been proposed. In the so-called multifunctional NPs, each single compound plays its role to achieve the maximum therapeutic performance. Such micelles can contain, at the same time, ligands and/or cell penetrating peptides on their surface for selective targeting and enhanced intracellular delivery of a combination of drugs and nucleic acids. In addition, a number of internal and external stimuli for triggered release of the drugs at disease sites have been added. Using this concept, Zhu et al. proposed multifunctional enzyme-sensitive micelles for the co-delivery of siRNA and paclitaxel [99]. In this study, a conjugate DOPE-PEI was linked to a PEG chain by introducing a matrix-metallo proteinase (MMP2)-sensitive linker, a tumoral-stimuli sensitive linker. Indeed, over-expressed levels of MMP2 and MMP9 have been found in the tumor microenvironment. They cause tumor invasion, progression, and metastasis of most human tumors by degrading the intercellular collagen matrix. In an aqueous environment, the so-called PEG-pp-PEI-PE conjugate formed “core-shell” micelles like-structures in which paclitaxel (PXL) and siRNA were co-incorporated. The system showed at the same time the capability to co-incorporate different active agents, excellent physical characteristics, and tumor targeting. In addition, the detachment of the PEG chain by the high levels of MMP2 in the tumor environment, expose the previously-hidden PEI to cells for better internalization of both siRNA and PXL. In vivo, in a non-small lung cancer carcinoma (NSCLC) xenograft mouse model, the stimuli-sensitive mixed micelles accumulated in tumor tissues and were efficiently internalized by tumor cells. A 2.4-fold higher internalization of the MMP2-sensitive formulation in tumor cells was achieved compared to the non-sensitive counterpart [99]. Despite the interesting results obtained in different experimental animal models of tumors, nowadays, clinical studies on micelles encapsulating ncRNA are not present on the clinical database [100], suggesting that further efforts are still required to bring lipid micelles from the bench to the bedside.

## 6. Lipid Nanovectors for ncRNA Delivery: The Clinical Trials

Despite, for many years, lipid nanocarriers being confined to laboratory use, at the moment different clinical trials have demonstrated the possibility to use these systems to deliver ncRNA in humans. Clinical trials based on RNAi and involving the use of formulations based on lipid vesicles are summarized in Table 2. In this context, the study NCT01262235 is in the most advanced stage. It has been designed to determine the safety, tolerability, pharmacokinetics, and pharmacodynamics of SNALPs encapsulating siRNA (TKM-08030) in adult patients with solid tumor or lymphomas that are refractory to standard therapies or for whom there is no standard therapy [100]. This study has completed the phase I and II, although the results are not yet available. Clinical trials involving lipoplexes containing RNA oligonucleotides are at the starting blocks within the Mutanome Engineered RNA Immuno-Therapy (MERIT) project, an initiative that received research funding from the European Union, coordinated by BioNTech AG. CLs to form RNA-lipoplexes, namely MERIT-Lipo (ClinicalTrials.gov Identifier: NCT02410733), have been chosen for the clinical trial titled “Evaluation the safety and tolerability of i.v. administration of a cancer vaccine in patients with advanced melanoma (Lipo-MERIT)”. The Lipo-MERIT vaccine consists of cationic liposomes with four naked ribonucleic acid (RNA)-drug products (DPs) RBL001.1, RBL002.2, RBL003.1, and RBL004.1, whihc are optimized to induce antigen-specific CD8+ and CD4+ T cell responses against the four selected malignant melanoma-associated antigens, respectively. This study is currently recruiting participants for the clinical phase I of the trial and the first outcome data are expected on January 2017.

Another clinical study (ClinicalTrials.gov Identifier: NCT01829971), [100] involves the use of a liposomal formulation for the delivery of miR-34a in patients with primary liver cancer or other selected solid tumors or hematologic malignancies, such as small cell lung carcinoma, lymphoma, melanoma, multiple myeloma, renal cell carcinoma, and non-small cell lung carcinoma. Moreover, the company Mirna Therapeutics developed a planned therapy based on lipid-nanovectors, i.e., SMARTICLES^®^ for the delivery of miR-34a in cancer. SMARTICLES^®^ are liposomes with amphoteric lipids that offered the possibility to be positively charged at acid pH and anionic at higher pH. This peculiarity allowed a high encapsulation of miRNAs, while the anionic charge present on the surface of the vectors at physiological pH prevents vesicle aggregation. Many studies have already reported promising results with the treatment of vectors encapsulating miR-34a in an orthotopic model of hepatocellular carcinoma [101,102,103]. Nowadays miR-34a-SMARTICLES^®^, namely MRX34, are under clinical evaluation (phase I). 

NCT02110563 is a phase 1 study to assess the safety and tolerability of the anticancer drug DCR-MYC. This formulation is based on a lipid nanoparticle suspension containing a novel synthetic double-stranded RNA (siRNA) against the activation of oncogene MYC involved in the growth of many hematologic and solid tumor malignancies, such as solid tumors, multiple myeloma, non-Hodgkins lymphoma, and pancreatic neuroendocrine tumors. In this study, the nanocarrier is reported as “stable lipid particles”, presumably a technology close to the SNALPs. This study is currently recruiting participants. The same company organized a phase I clinical trial study (NCT02314052) focused on the investigation of the same anticancer drug (DCR-MYC) on patients with hepatocellular carcinoma. Final data collection is scheduled for October 2016. 

Although any product is, at the moment, approved for clinical use, there is a growing number of clinical studies, suggesting that lipid vesicles could soon enter into clinical practice, paving the way for the therapeutic use of ncRNA for cancer.

## 7. Conclusions

The use of lipid nanovectors to deliver nucleic acids is not recent and their potential as transfection agents is well established. However, the instability of lipid nanovectors in the biological environment, and the variability of the delivery efficiency, have limited for many years their in vivo application. However, the large number of research studies in the field have been focused on optimizing RNA transfection efficiency, as well as to limit instability in vivo and rapid clearance. In recent years, more clinical trials focused on the use of siRNA and miRNA as new therapeutics, and involving lipid-based nanovector as delivery systems, started. Although further optimization of the lipid nanovectors is still possible, we are certainly very close to the use of these formulations in therapy, thus opening new perspectives for cancer therapy. Moreover, due to the possibility to easily modify the lipid particle with targeting moieties, and taking into account the promising results obtained so far, more selective RNA-based therapy will be possible in the not-too-distant future. 

## Figures and Tables

**Table 1:** Summary table of the most meaningful findings in ncRNA delivery with lipid vesicles.

Nanocarrier Composition	Findings	Reference
DOTMA/DOPE-CLs	Lipid/RNA weight ratio of 2.5 showed the highest RNA transfection (70% of the transfected cells)	[24]
DC-chol/DOPE-CLs	The highest siRNA transfection efficiency were found at the DC-chol/RNA weight ratio of 5 or 10 and at a DC-chol/DOPE molar ratio of 1	[25]
DOTAP/DMPG-CLs	DMPG was found to neutralize the net surface charge of CLs reducing the cytotoxicity and the RNA complexation efficiency	[27]
DOTAP/HSPC-CLs	HSPC increased the complexation strength between DOTAP liposomes and siRNA	[28]
TEPA-PCL-CLs	The proton sponge effect of the polycation lipid TEPA-PCL enhanced the cellular-uptake and endosomal escape of miR-92a	[38]
DOPE/chol/DCP-DETA-CLs	The inclusion of polycationic lipid DCP-DETA in liposomes increased the biological stability of encapsulated siRNA compared to conventional CLs	[38]
DOTMA/DOPE-CLs	Pre-incubation of CLs with phosphate buffer reduced the time for RNA transfection	[40]
DOPE-CLs	Agitation during siRNA/CLs complex formation increased the complexation efficiency and gene knockdown	[41]
DOTMA-CLs	DOTMA is more cytotoxic than DOTAP due to the more stable ether linker	[42]
NaChol/DOTAP-CLs	DOTAP/NaChol at the weight ratio of 8:1 and siRNA at a RNA/CL weight ratio of 16:1 allowed to achieve the highest permeation through the and highest siRNA internalization into melanoma UACC-903 cells	[51]
DOTMA/chol/D-Alpha-tocopheryl-PEG succinate-CLs	The use of chol as helper lipid increased the RNA delivery into the lungs, and reduced the RNA delivery in other organs, e.g., into the liver	[60]
DOTMA/OA/PEGylated chol-CLs	The use of OA as helper lipid changed lipoplex biodistribution improving miR-122 level in liver in an experimental model of liver cancer, reducing toxicity in non-target organs	[61]
DODAP-SNALPs	The ionizable lipid DODAP improved RNA encapsulation efficiency; the possibility to neutralize the charge after RNA encapsulation enhanced the vesicle stability in biological fluids	[66]
DLinDMA-SNALPs	The highest number of double bonds of DLinDMA reduced the phase transition temperature with a significant improvement of the transfection efficiency	[66]

**Table 2:** Clinical trials involving lipid vesicles encapsulating ncRNA for cancer treatment.

Clinical Trials					
Clinical Trials Identifier	Lipid Nanovector	ncRNA	Condition	Administration Route	Companies
NCT01591356	Neutral liposome	siRNA	Advanced Cancers	intravenous	M.D. Anderson Cancer Center
NCT01262235	SNALP	RNA	Neuroendocrine Tumors; Adrenocortical Carcinoma	intravenous	Arbutus Biopharma Corporation
NCT02410733	SNALP	RNA	Melanoma	intravenous	Biontech RNA Pharmaceuticals GmbH
NCT01829971	SMARTICLES^®^	mRNA	Primary Liver Cancer; Lymphoma; Melanoma; Multiple Myeloma; Renal Cell Carcinoma	intravenous	Mirna Therapeutics, Inc.
NCT02110563	Stable lipid particles *	siRNA	Solid Tumors; Multiple Myeloma; Non-Hodgkins Lymphoma; Pancreatic Neuroendocrine Tumors	intravenous infusion	Dicerna Pharmaceuticals, Inc.
NCT02314052	Stable lipid particles *	siRNA	Hepatocellular Carcinoma	intravenous infusion	Dicerna Pharmaceuticals, Inc.

* As referred in the study record (ClinicalTrials.gov).

## Abbreviations

RNAi: RNA interference; miRNA: micro RNA; siRNA: small interfering RNA; ncRNA: non-coding RNA; DTPA: diethylenetriaminepentaacetic acid; EPR: enhanced permeability and retention effect; RES: reticuloendothelial system; PEG: polyethylene glycol; CLs: cationic liposomes; DOTMA: *N*-[1-(2,3-dioleyloxy)propyl]-*N*,*N*,*N*-trimethylammonium chloride; DOTAP: 1,2-dioleoyl-3-trimethylammonium-propane (chloride salt); DOGS: dioctadecylamidoglycylspermine; DC-chol: 3β-[*N*′,(*N*′,*N*′-dimethylaminoethane)-carbamoyl] cholesterol; DOSPA: 2,3-dioleyloxy-*N*-[2(sperminecarboxamido)ethyl]-*N*,*N*-dimethyl-l-propanaminium trifluoroacetate; chol: cholesterol; DOPE: dioleoylphosphatidyl ethanolamine; HSPC: hydrogenated soya phosphocholine; OA: oleic acid; TEPA-PCL: polycation dicetyl phosphate-tetraethylenepentamine; HUVECs: human umbilical vein endothelial cells; DCP-DETA: polycation dicetyl phosphate-diethylenetriamine; HOS: human osteosarcoma cell line; PKC: protein kinase C; DMPG: dimyristoyl-*sn*-glycero-3-phosphoglycerol; NaChol: sodium cholate; NSCLC: non-small–cell lung cancer; DSPE-PEG: 1,2-distearoyl-sn-glycero-3-phosphoethanolamine-*N*-[amino(polyethylene glycol)-2000]; PDAC: adenocarcinoma cells; SNALPs: stable nucleic acid lipid particles; DSPC: 1,2-distearoyl-sn-glycero-3-phosphocholine; DODMA: 1,2-dioleyloxy-N,N-dimethyl-3-aminopropane; DODAP: 1,2-dioleyl-3-dimethylammonium propane; DSDMA: 1,2-distearyloxy-*N*,*N*-dimethyl-3-aminopropane; DLinDMA: 1,2-dilinoleyloxy-*N*,*N*-dimethyl-3-aminopropane; DLenDMA: 1,2-dilinolenyloxy-*N*,*N*-dimethyl-3-aminopropane; MERIT: Mutanome Engineered RNA Immuno-Therapy; RNA: ribonucleic acid; DPs: drug products; LPD NPs: liposome-polycation-DNA nanoparticles; DOX: doxorubicin; HA: hyaluronic acid; LPH NPs: hyaluronic acid nanoparticles; scFv: antibody fragment; CaP NPs: calcium/phosphate nanoparticles; LNPs: lipid-based nanoparticles; Han: hyaluronan; SLNs: solid lipid NPs; PTX: paclitaxel; PEI: polyethylenimine; PEC: polyelectrolyte complex; PE: PEG derivative with lipids; MCF7/MDR: multi-drug resistant breast cancer cells; GFP: green fluorescent protein; GSH: including glutathione; MMP2: matrix-metallo proteinase.
